# Human adipose-derived stem cells cultured in keratinocyte serum free medium: Donor’s age does not affect the proliferation and differentiation capacities

**DOI:** 10.1186/1423-0127-20-59

**Published:** 2013-08-14

**Authors:** Dah-Ching Ding, Hsiang-Lan Chou, Wei-Ting Hung, Hwan-Wun Liu, Tang-Yuan Chu

**Affiliations:** 1 Department of Obstetrics and Gynecology, Buddhist Tzu Chi General Hospital; Tzu Chi University, Hualien, Taiwan; 2 Institute of Medical Sciences, Tzu Chi University, Hualien, Taiwan; 3 Stem Cell Laboratory, Department of Research, Buddhist Tzu Chi General Hospital, Hualien, Taiwan; 4 Department of Occupational Medicine, Buddhist Tzu Chi General Hospital, Tzu Chi University, Hualien, Taiwan

**Keywords:** Adipose-derived stem cells, Donor’s age, Proliferation, Differentiation, Telomere length

## Abstract

**Background:**

Although donor age-related effects of characteristics of mesenchymal stem cells (MSC), such as a decrease in the proliferation and differentiation capacity and an increase of senescence and apoptosis, are evident, such effects are generally less prominent in adipose-derived stem cells (ASC). Using a hormone and growth factor rich medium (KFSM), this study cultured ASC from abdominal subcutaneous fat of 27 adult females in three age groups: 30-39 y, 40-49 y and 50-60 y, and investigated the growth and differentiation characteristics.

**Results:**

The derived ASC had an immunophenotype similar to that of bone marrow derived MSC (BMSC). They could be stably expanded with an average population doubling time of 21.5 ± 2.3 h. Other than a higher pre-adipogenic commitment and a lower adipogenic differentiation capability in ASC derived from the old age group, other characteristics including proliferation rate, doubling time, telomere length, as well as the osteogenic and chondrogenic differentiation capacity were the same regardless of the donor’s age.

**Conclusions:**

The study demonstrates a promising proliferation and differentiation capabilities of ASC regardless of the donor’s age. The compromised adipogenic potential in the older donors could be a benefit for their application in regeneration therapy.

## Background

Aging is a complex process characterized by a variety of disorders associated with generalized decline and incapability to maintain tissue homeostasis. Aging tissues typically demonstrate increased preponderance for degenerative disorders and decreased repair capacity [1]. Stem cells in numerous tissues replace mature cell loss during physical activity or injury throughout life. The role of stem cells in aging as well as age related decline in function of stem cells are hotly debated issues [2,3].

Nowadays, increasing evidence supports the hypothesis that cellular senescence recapitulates aspects of organism aging and contributes to aging phenotypes in vivo [4,5], in part by limiting self-renewal of tissues by progenitor cells [6,7]. The senescent phenotype is characterized by features such as enlarged cell size, flattened morphology, and enhanced senescence-associated (SA)-β-galactosidase activity. Of the same concern is the senescence potential of mesenchymal stem cells (MSC) derived from old donor. MSC has widely being implicated as source for cell therapy in regenerative medicine. For clinical application of MSC, a long term proliferation capacity without senescence is important and requires a long telomere. Telomeres protect chromosome ends from wasting by repeated cell division [8] and are regarded as the mitotic clock of the cell replication capacity [9,10].

MSCs have been characterized in tissues including bone marrow, adipose, skeletal muscle, dermis and umbilical cord [11] with similar morphological and immunophenotypical characteristics [12]. Adult tissue-derived MSC, such as bone marrow derived MSC (BMSC) [13], have attracted a lot of attention in cell therapy. However, usage of BMSC may be restricted by donor age-dependent decline of capability of proliferation [14-16]. Besides, the osteogenic potential of BMSC has been reported to be compromised with the advanced age of donor [17,18].

An alternative source MSC is adipose-derived stem cells (ASC), which are thought to have advantages over BMSC for the high abundance of source and ease of isolation, expansion and cryopreservation [19]. The effect of donor’s age on the proliferation and differentiation of ASC is largely controversial (Table 1). There are claims that old age of donor does not seem to affect the viability of ASC [20,21] but other reports showed a compromised viability [3,22,23]. While most reports showed a lower capacity of adipogenic differentiation for old age donor [3,21,22,24] but one report showed no difference [23]. As to osteogenic differentiation, there were reports of lower [3,24], higher [23] and no different [20] capacity in ASC from old donor.

**Table 1:** Summary of age-related characteristics of ASC in different studies

**Study**	**No. of donors**	**Age range**	**Culture condition**	**Proliferation**	**Osteo-genesis**	**Adipo-genesis**	**Chondro-genesis**	**Origin of ASC**
Van Harmelen et al. 2004 [22]	29	17-61	DMEM/F12 + 10%FCS	**↓**		**↓**		Abdominal subcutaneous fat and omental fat
de Girolamo et al. 2009 [24]	26	21-68	DMEM + 10%FBS		**↓**	**↓**		Abdominal subcutaneous fat
Zhu et al. 2009 [23]	26	20-58	DMEM + 10%FBS	**→**	**↑**	**→**		Abdominal subcutaneous fat
Alt et al. 2012 [3]	40	15-71	α-MEM + 20% FBS	**↓**	**↓**	**↓**	**↓**	Abdominal subcutaneous fat
Chen et al. 2012 [20]	22	36-71	KFSM + NAC + 5% FBS	**→**	**→**			Gluteal fat
Present study	27	30-60	KFSM + NAC + 5% FBS	**→**	**→**	**↓**	**→**	Abdominal subcutaneous fat

*NAC* N-acetyl cysteine.

*KFSM* keratinocyte serum free medium.

*FBS* fetal bovine serum.

*FCS* fetal calf serum.

We assume a medium of low calcium and low serum with hormone and antioxidant supplements such as the keratinocyte serum free medium (KSFM) may overcome the donor-age effect of ASC, thus explain the controversy of previous studies. A comprehensive characterization of the growth and differentiation properties as well as senescence and telomere length were done in KSFM-cultured ASC from donors of different ages. The results showed a comparable growth and non-adipogenic differentiation capacities in ASC regardless of the donor’s age.

## Methods

### Source of adipose tissue, patient age groups, and body mass index

Twenty-seven female donors who underwent gynecological surgery were enrolled. The subjects were classified into three age groups: 30–39 y (n = 10), 40–49 y (n = 10) and 50–60 y (n = 7), with mean age of 33.8 ± 3.7, 42.5 ± 2.7 and 51.8 ± 3.6 y, respectively. The body mass index (BMI) was calculated as body weight (kg)/height (m^2^). The mean BMI in each group was 26.1 ± 5.3, 25.4 ± 4.8 and 25.3 ± 2.5 kg/m^2^, respectively. The Research and Ethics Committee of Buddhist Tzu Chi General Hospital approved this study, and informed consent was obtained from each subject prior to tissue collection.

### Derivation of ASC

Human adipose tissue was harvested from subcutaneous fat (1 cm^3^) from the abdominal wall during gynecologic surgery. Tissue samples were placed in Ca^2+^/Mg^2+^-free phosphate-buffered saline solution (PBS), and then transferred to the laboratory immediately. Human adipose tissue was removed from the transport medium, placed in a Petri dish, and cut into small pieces (1–2 mm^3^) in the presence of Ca^2+^/Mg^2+^-free PBS. Tissues were dissociated with 0.1 mg collagenase Ia (Sigma, St. Louis, MO, USA) and incubated for 60 min at 37°C. Following enzymatic digestion, the resulting cells were collected and cultured in keratinocyte-serum-free medium (KSFM) (added epidermal growth factor and bovine pituitary extract, Gibco, 17005-042, USA) with 5% fetal bovine serum (FBS), N-acetyl cysteine (NAC), L-ascorbic acid-2-phosphate. Supernatant and debris were removed from the culture dish on day 2 of culturing. The resulting ASC culture was denoted as passage 0. To prevent spontaneous differentiation, cultures were maintained at sub-confluent levels (<80% confluency). We usually passaged cells at a ratio of 1:3. Passaging of ASC cultures was performed using 2.5% trypsin/0.23 mM ethylenediaminetetraacetic acid (EDTA). Passaged cultures were defined as passage 1.

### Flow cytometry of ASC

Surface molecules of ASC cultures of passage 3 were characterized by flow cytometry. Cells were detached with 2 mM EDTA in PBS, washed with PBS containing 2% bovine serum albumin (BSA) and 0.1% sodium azide (Sigma, USA), and incubated with their respective antibody conjugated with fluorescein isothiocyanate (FITC) or phycoerythrin (PE), including clustering of differentiation (CD)13, CD34, CD44, CD45, CD56, CD90 and human leukocyte antigen (HLA)-ABC (BD, PharMingen, Franklin Lakes, NJ, USA). Cells were analyzed using a flow cytometer (Becton Dickinson, San Jose, CA, USA).

### Proliferation assay and estimation of population doubling time

The ASC were seeded in triplicate at a density of 2 × 10^3^ cells/cm^2^, in a 96-well plate with KSFM with 5% FBS. ASC at passage 2-3 were used for proliferation assay. Cells were harvested and counted using a cell proliferation kit (XTT based, Biological Industries Ltd., Kibbustz Beit Haemek, Israel) on days 0, 2, 3 and 4, and a growth curve was generated. XTT solutions and PMS (N-methyl dibenzopyrazine methyl sulfate) were defrosted immediately prior to use in a 37°C bath. PMS was added to the XTT solution immediately before use. 50 μl of XTT/PMS was added to each 100 μl culture. After 2-5 h of incubation at 37°C, the optical density (OD) of the wells was determined using a spectrophotometer (ELISA reader) at a wavelength of 450 nm and a reference wavelength of 650 nm. To calculate the population DT, 1 × 10^4^ cells were seeded in a 10-cm Petri dish. The KSFM was changed on day 4 and cells were harvested and counted on day 7. The DT was calculated according to the formula: DT = log (final cell number)-log (initial cell number) = K × T, where K is the generation constant (0.008963) and T is time in hours [25].

### Adipogenesis and measurement

Passage 2-3 of ASC were seeded in a 12-well plate at a density of 5 × 10^4^ with adipogenic medium Dulbecco’s Modified Eagle Medium (DMEM) supplemented with 10% FBS, 1 μmol/L dexamethasone, 5 μg/mL insulin, 0.5 mmol/L isobutylmethylxanthine and 60 μmol/L indomethacin). These ASC were allowed to grow for 14 days. The medium was changed every 3 days, after which, the ASC were stained with Oil Red O. After staining, the samples were washed twice with PBS. The lipids were then extracted from the cells by 100% isopropanol and gentle shaking for 5 min. The concentration of the lipids was measured based on the absorbance at 510 nm. The lipid quantity for each sample was measured in triplicate.

### Osteogenesis and measurement

The passage 2-3 of ASC were seeded in a 12-well plate at a density of 1 × 10^4^ and grown with osteogenic medium (DMEM supplemented with 10% FBS, 0.1 μmol/L dexamethasone, 10 mmol/L β-glycerol phosphate, and 50 μmol/L ascorbate) that was changed every 3 days. Cells were allowed to grow for 21 days and stained with Alizarin Red. For quantification of staining, 800 uL 10% (v/v) acetic acid was added to each well, and the plate was incubated at room temperature for 30 min with shaking. The monolayer, now loosely attached to the plate, was then scraped from the plate with a cell scraper (Fisher Scientific, Hampton, NH, USA) and transferred with 10% (v/v) acetic acid to a 1.5-mL microcentrifuge tube with a wide-mouth pipette. After vortexing for 30 s, the slurry was overlaid with 500 uL of mineral oil (Sigma-Aldrich), heated to exactly 85°C for 10 min, and transferred to ice for 5 min. The slurry was then centrifuged at 20,000 g for 15 min, and 500 uL of the supernatant was removed to a new 1.5-mL microcentrifuge tube. Then 200 uL of 10% (v/v) ammonium hydroxide was added to neutralize the acid. Aliquots (150 uL) of the supernatant were read in triplicate at 405 nm in a 96-well format using opaque-walled, transparent-bottomed plates (Fisher Scientific, Hampton, NH, USA).

#### Chondrogenesis and measurement

The passage 2-3 of ASC were seeded in a 12-well plate at number of 1 × 10^5^ cells and were grown in chondrogenic media consisting of DMEM, 10% FBS, 10 ng/ml TGF-β1, 50 μg/ml of ascorbic acid-2-phospate and 6.25 μg/ml of insulin, and media were changed every three days. Cells were incubated with the chondrogenic media at 37°C with 5% CO_2_ for three weeks. After fixing in paraformaldehyde, cells were mounted on slides and stained using standard Alcian Blue protocols. The cells also processed for processed gene expression analysis via quantitative reverse transcription polymerase chain reaction (qRT-PCR) for cartilage specific matrix gene expression at Day 0 and Day 21. For quantification of incorporation of Alcian blue into the proteoglycan-rich extracellular matrix, cultures were incubated with 6 M guanidine hydrochloride overnight, and subjected to photometric measurement at optical density (OD) 595 nm [26]. For chondrogenesis in pellet culture, a total of 5 × 10^5^ cells were spun in 15 ml sterile conical polypropylene tubes (Enzymax LLC, Kentucky, USA) at 1000 rpm for five minutes to form spherical cell pellets. The final volume of chondrogenic media was 250 μl per pellet. Medium change was performed three times a week. The pellets were cultured for three weeks to allow appreciable matrix accumulation. Thereafter, the pellets were measured their sizes using ImageJ software (free software developed by NIH).

### RNA isolation and reverse transcription-polymerase chain reaction (RT-PCR)

Total RNA was extracted using RNEasy® (Qiagen, USA) according to the manufacturer’s instructions. Reverse transcription-polymerase chain reaction (RT-PCR) with specific primers was performed as described previously [27]. Briefly, total RNA was collected using TRIzol (Invitrogen, Carlsbad, CA, USA), and complementary DNA was synthesized using a SuperScript first-strand synthesis system (Invitrogen, Carlsbad, CA, USA). Complementary DNA was amplified by PCR using the AmpliTaq Gold Kit (Applied Biosystems, Foster City, CA, USA). The PCR products were resolved on 2% agarose gels.

### Real-time quantitative RT-PCR (qRT-PCR)

Real time quantitative RT-PCR was performed using TaqMan Gene Expression assays and Applied Biosystems (ABI) Step One Plus (Applied Biosystems, USA). Primer sequences for the adipogenesis gene, *peroxisome proliferator-activated receptor* (*PPAR-γ)*, were 5′-AGC CTC ATG AAG AGC CTT CCA-3′, 5′- TCC GGA AGA AAC CCT TGC A-3′; for the osteogenesis genes, *osteopontin* were 5′- AGG AGG AGG CAG AGC ACA-3′, 5′- CTG GTA TGG CAC AGG TGA TG-3′; for the chondrogenisis genes, *collagen type 2A1* (*COL2A1*) and *aggrecan* (*ACAN*) were: 5'-CAA CAC TGC CAA CGT CCA GAT-3', 5'-TCT TGC AGT GGT AGG TGA TGT TCT-3'; and 5'-ACA GCT GGG GAC ATT AGT GG-3', 5'-GTG GAA TGC AGA GGT GGT TT-3', respectively; and for the internal control, *glyceraldehyde 3-phosphate dehydrogenase* (*GAPDH)*, were 5′- GGC AGC AGC AAG CAT TCC T-3′, 5′- GCC CAA CAC CCC CAG TCA-3′. The PCR conditions were as follows: initial incubation at 50°C for 2 min and denaturation at 95°C for 10 min, followed by 40 cycles of 95°C for 15 sec and 60°C for 1 min.

### Analysis of SA β-Gal activity

Senescence-associated β-galactosidase (SA-β-Gal) is a commonly used marker for cell senescence [28,29]. For SA-β-Gal stain, the ASC were washed with PBS, fixed for 3-5 min (at room temperature) in 1% paraformaldehyde, washed and incubated at 37°C (no CO2) with fresh β-Gal stain solution (Cell Signaling Technology, Beverly, MA, USA). Semiquantitative measurement of SA β-Gal staining was performed by calculating the number of β-Gal positive cells in three low power fields (100×) and expressed as a percentage of all counted cells.

### Measurement of telomere length by real time qPCR

ASC at passage 4 were used for telomere length assay. Real-time qPCR assay of telomere length [30] was followed with minor modifications. Two real time PCRs were performed, one to determine the cycle threshold (Ct) value for telomere amplification, and the other to determine the Ct value for amplification of a single-copy control gene *RPLP0* (*acidic ribosomal protein P0*), with primer sequences and PCR conditions described in [30]. All real-time qPCRs were carried out using the ABI Step One Plus Sequence Detection System (Applied Biosystems). Intra- and inter-assay reproducibility of both telomere and *RPL0* PCR results were evaluated initially in a series of experiments using dilutions. The standard deviation (% of coefficient of variation) of Ct values in 3 replicates of samples amplified in the same PCR run for telomere and *RPL0* were ≤ 0.16 (≤0.73%) and ≤0.22 (≤0.50%), respectively. Both reference DNA and DNA samples were analyzed in duplicate. Mean Ct values were used to calculate relative telomere length using the telomere to sample (T/S) ratio derived from the following formula: ΔCt_sample_ = ΔCt_telomere_ − Ct_control_, ΔΔCt = ΔCt_sample_ − ΔCt_reference curve_ (where ΔCt_reference curve_ = Ct_telomere_ − Ct_control_) and then T/S = 2^−^^ΔΔCt^ .

### Statistics

GraphPad Prism version 5.00 for Windows was used in this study. Mean levels of differentiation (adipogenesis and osteogenesis) and gene expression (PPAR-γ and osteopontin) across three donor age groups were compared using one way ANOVA and post-hoc tests (the Bonferroni multiple comparison test). The level of significance was set at 0.05.

## Results

### ASC has an immunophenotype similar to BMSC

**Figure 1:**
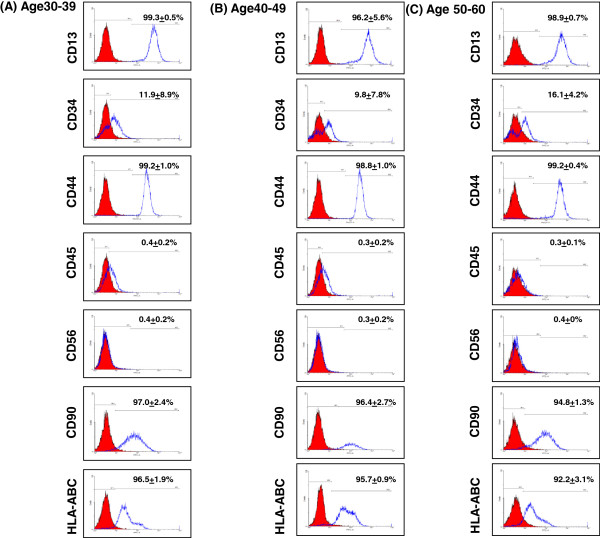
**Immunophenotyping of ASC derived from donors of three age groups.** Histograms of cell surface markers are demonstrated in ASC derived from three age groups (n = 3 in each group) at passage 3. The respective isotype controls are showed as a shaded histograms. ASC from the three age groups were all positive for CD13, CD34, CD44, CD90 and HLA-ABC, and negative for CD45 and CD56.

The derived ASC at passage 2 to 3 had a fibroblast-like appearance resembling that of BMSC [31]. These ASC in initial cultures were homogeneous in appearance and could reach 80-90% confluence by 14 days. This morphology was maintained up to passage 22. Flow cytometry revealed surface expressions of CD13, CD44, CD90 and HLA-ABC (typical MSC markers), while white blood cell and NK cell markers CD45 and CD56 were not expressed (Figure 1). This pattern is consistent with that of BMSC [31]. Meanwhile, CD34 was expressed in 11.9 ± 8.8%, 9.8 ± 7.7% and 16 ± 4% of ASC derived from 30-39 y, 40-49 y and 50-60 y age groups, respectively (*P* = 0.59, ANOVA test). Previous report has revealed CD34 + cells can be found in the ASC [32].

### Proliferation rate and doubling time of ASC were not compromised in older donor and low BMI

**Figure 2:**
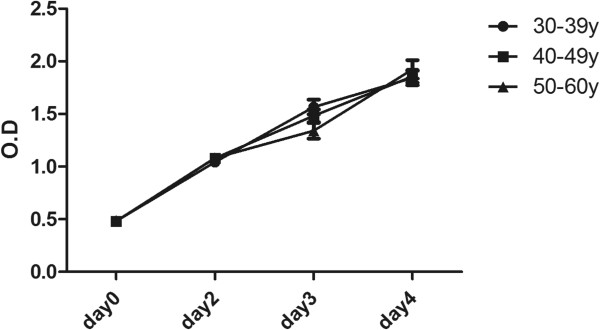
**Growth kinetics of ASC derived from the three age groups.** Growth kinetics, as indicated as mean ± SD of the OD read of cell density, of the three age groups are showed. The case number of the 30-39 y, 40-49 y and over 50 y groups was 10, 10 and 7, respectively. * *P* < 0.05.

**Figure 3:**
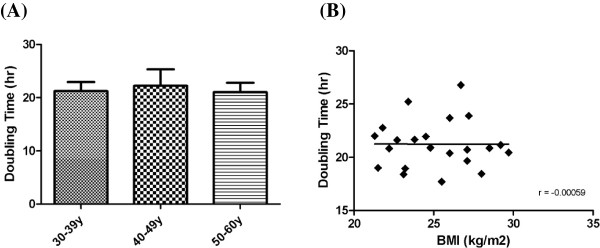
**The population doubling time of ASC in relation to age and BMI of donors.** The doubling times of cultured ASC from the three age groups (n = 7, 9 and 5 in the 30-39 y, 40-49 y and 50-60 y groups, respectively) **(A)** and donor’s BMI **(B)** (n = 22) were compared.

Growth curves of the first passage of ASC cultures of different donor age groups are outlined in Figure 2. The average population doubling time (DT) for all ASC donors was 21.5 ± 2.3 h. No significant difference in growth kinetics (Figure 2) and DT (Figure 3A) existed among the three age groups. There was also no significant correlation of BMI of the donor and population DT of derived ASC (Figure 3B).

### Lower adipogenic potential of ASC in the old age groups

**Figure 4:**
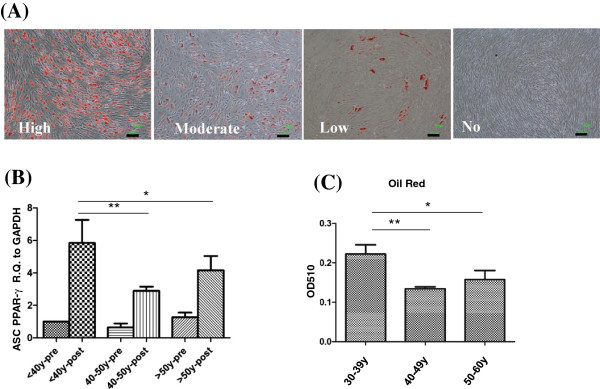
**Compromised of adipogenic differentiation capability in ASC derived from old donors. (A)** Different levels of Oil Red O staining after induction of adipogenic differentionesis were demonstrated. Scale bar = 100 μm. **(B)** Mean mRNA levels of *PPAR-γ* are compared in different donor’s age groups (n = 3 in each group). Levels are expressed as mean ± SD. Pre: pre-induction. Post: post-induction. **(C)** Quantification of Oil Red O expression of ASC in the three groups (n = 3 in each group). *P < 0.05, ** *P* < 0.01.

Adipogenesis and lipid vacuole formation in the ASC were studied by staining cells with Oil Red O. At day 14 post adipogenic inductions, ASC contained large Oil Red O-positive lipid droplets within their cytoplasm (Figure 4A). The amount of both intracellular lipid and the expression of *PPAR-γ* gene were lower in the 40-49 y and ≥50 y age groups than in the 30-39 y group (*P* for both markers were <0.01 and <0.05, respectively) (Figure 4B and 4C).

### Osteogenic potential of ASC was not related to donor’s age

**Figure 5:**
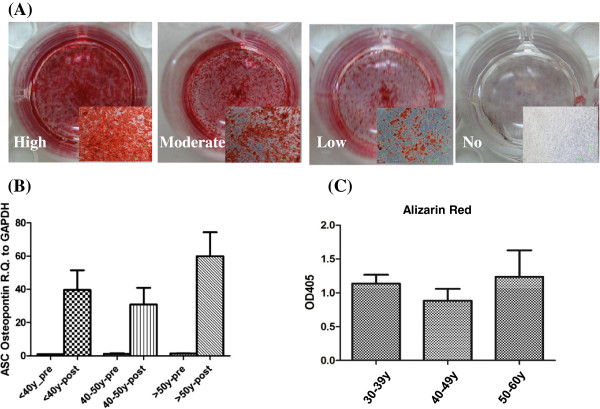
**No difference of osteogenic differentiation in ASC derived from different age groups.** Parameters of osteogenic differentiation including Alizarin Red staining **(A)**, *Osteopontin* mRNA **(B)** and quantification of Alizarin Red **(C)** were compared in the ASC derived from three age groups (n = 3 in each group). Levels are expressed as mean ± SD. Pre: pre-induction. Post: post-induction.

Previously, a decline of osteogenic potential of ASC derived from old donors was reported [14]. We performed a detail characterization of osteogenesis of ASC. Osteogenesis in ASC was stained with Alizarin Red to determine calcium deposition. The cellular morphology changed from spindle shape to cuboid shape. Low level of osteogenesis were characterized by formation of a monolayer of Alizarin Red–positive cells, while higher staining levels were characterized by the presence of strongly stained multi-layered Alizarin Red–positive nodular structures with well-defined inter-nodular regions not containing cells (Figure 5A). Osteogenic levels as measured by quantified Alizarin Red staining and expression of *Osteopontin* were not different among the three age groups (Figure 5B and 5C).

### Chondrogenic potential was not related to donor’s age

**Figure 6:**
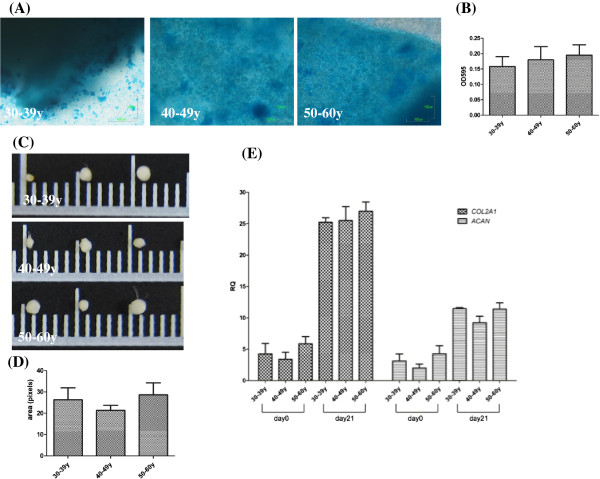
**No difference of chondrogenic differentiation in ASC derived from different age groups.** Parameters of chondrogenic differentiation including Alcian blue staining **(A)**, quantification of Alcian blue at OD 595 nm **(B)**, micromass formation **(C)**, size of micromass **(D)** and *COL2A1* (Collagen type 2A1) and *ACAN* (Aggrecan) mRNA level **(E)** were compared in ASC derived from the three age groups (n = 3 each). Levels are expressed as mean ± SD.

Upon induction of chondrogenic differentiation, ASC conglobulated to micromass (Figure 6C). The sizes of the microsphere as well as the levels of Alcian blue staining of chondrogenic proteoglycan (Figure 6A) and expression of chondrogenic genes, *COL2A1* and *ACAN*, were not different among the three age groups (Figure 6B-6E).

### Senescence and telomere length of ASC

**Figure 7:**
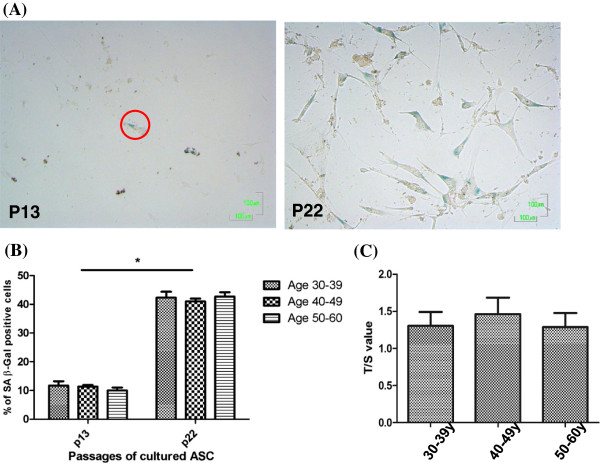
**No difference of senescence-associated β-Gal activity and telomere length in ASC derived from different age groups.** Representative pictures **(A)** and the quantitative measurement **(B)** of senescence-associated (SA) β-Gal staining in ASC derived from three age groups (n = 5 each) at two different passages are showed. *P < 0.05. **(C)** Relative lengths of telomere, as expressed as telomere/single-copy-gene (T/S) ratios, are showed (n = 10 each in 30-39 y and 40-49 y groups, n = 7 in 50-60 y group).

Senescent ASC express SA β-Gal to form a local blue precipitate (Figure 7A). There was a significant increase in the numbers of SA β-Gal-positive cells in the ASC cultured in late passage (P22) than the early passage (P13) (Figure 7B); but the proportion of senescent cells in the ASC culture did not differ among different age groups in the same passage (Figure 7B). As shown in Figure 7C, there was no significant difference in the telomere length of ASC derived from the three age groups at passage of 4.

## Discussion

The result of this study revealed that donor’s age does not affect the capability of osteogenesis and chondrogenesis of ASC, but the capability of adipogenenic differentiation is significantly compromised in the old age donors. There were significant lower levels of *PPARγ* mRNA and Oil Red staining upon induction of adipogenic differentiation. PPARγ is regarded as an adipogenic transcription factor. Decrease of PPARγ could contribute to the age-related declines in fat cell size and the capacity to store lipid as well as insulin responsiveness [33]. PPARγ is lower in preadipocyte cultured from older than younger human following exposure to differentiation medium [21,34]. PPARγ is also reduced in fat tissue from various species in old age, including rats and primates [34,35]. In aged mice, an altered metabolism and volume of adipose tissue depots was observed. The weight of the brown, epididymal, inguinal, and retroperitoneal adipose depots were reduced by advanced age when total body weight was not changed [36].

There have been several reports of donor age effect on the characteristics of ASC. As summarized in Table 1, a wide range of variations of results of proliferation, osteogenesis and adipogenesis in relation to donor’s age was noted in these studies. The wide discrepancy can be due to differences of the gender of donor, range of age stratification, sources of the adipose tissue and culture conditions. There has been a report showing ASC derived from women are less capable of osteoblastic differentiation than ASC from men [37]. The age-related difference of osteogenesis can thus be less obvious in this study of women than others of both sexes. Subjects in the present study have an age range of 30 to 60 years. The result of lower adipogenesis of ASC derived from old age group is consistent with two previous studies with a wider range of age (15-71 years and 21-68 years) [3,24]. But we did not see the compromised osteogeneis in old age group as compared to the very young age group in these two previous reports.

We assume the use of different culture medium may be the main reason of difference. The present study used KSFM for culture of ASC. This is in contrast to the alpha-MEM [3], DMEM [23,38], or DMEM/F12 [22] used in other studies. These media contain a relative higher calcium concentration. High calcium content (DMEM) (1.8 mM) has been reported to adversely influence the growth and proliferation potentials of adipogenic cell [19,25,39]. KSFM medium is an optimized MCDB-153 medium with a low calcium content (0.09 mM). Supplementation with hormones (bovine pituitary extract), growth factors (rEGF) and anti-oxidants (NAC and ascorbic acid) can enhance the proliferation efficiency and lifespan of cells [25]. Interestingly, a previous study using this medium [20] showed the same results of no differences of proliferation capability in ASC derived from young (36.4 ± 11.8 years) and old (71.4 ± 3.6 years) donors (Table 1). Thus, the growth potentials of ASC derived from old donor could be maintained by culturing with low calcium and nutrient enriched medium such as KSFM.

In this study, around 10 ~ 16% of ASC were positive for CD34 surface marker. CD34 is often expressed on hematopoietic stem cells. Findings of CD34 expression in ASC have been inconsistent [40] and may relate to the duration of cell culture, since a progressive down-regulation of CD34 marker has been observed in ASC culture [41,42]. Loss of CD34 expression may be related to the physiological process of commitment and/or differentiation from an immature status to more differentiated ones such as the adipose, bone, or smooth muscle [32]. Conversely, the expression of CD34 may represent a preadipocyte commitment of ASC [40]. In this study, although there was no statistically significant difference of CD34 percentage among different age groups (*P* = 0.59), a seemly higher percentage of CD34 was found in ASC derived from the older age group (16.1% vs. 9.8% and 11.9%). ASC derived from older women may have a higher proportion of preadipocyte commitment and a lower capability of adipogenic differentiation.

Telomere shortening and resulted cell senescence and growth arrest can be the major hurdle of cellular therapy since it limits the expansion of MSC. It has also been concerned that the cell division clock may approach terminus in MSC from elderly donors. Using KFSM medium, this study revealed no difference of telomere length in ASC derived from the three age groups. This is in agreement with a previous report showing telomere length of early passage MSC does not correlate with the age of the donor [43].

For MSC of limited source such as bone marrow, extensive cell expansion may be needed for clinical use and may encounter cell senescence after long passage. As seen in this study, ASC is of no exception. A significantly higher SA-β-gal activity was found in late passages. In this regard, adipose tissue can be acquired in large amount to derive enough number of MSC for use without extensive expansion.

Previous studies have revealed an increase of cell doubling time with increased activity of SA-**β**-gal in BMSC derived from old donors [16]. Compared to BMSC, ASC has a generally lower activity of SA-**β**-gal, suggesting a less aging activity [16]. Although there was a significant correlation between the final population doubling number and the age of donors [42], we found an equivalent cell doubling time as well as SA-**β**-gal activity in ASC derived from different donor age groups at earlier passages up to p22. This amount of expansion is expected to generate enough ASC for most regenerative uses.

## Conclusions

In summary, this study demonstrates a promising proliferation and longevity capacity of ASC regardless of the age of the donor. Other than less adipogenesis, ASC from elder donors maintain the capability of osteogenic and chondrogenic differentiation and longevity. The autologous ASC from the elderly may become a promising therapeutic agent especially for tissue repair and the lower capacity of adipogenesis may be a benefit in this utilization since adipose tissue is generally not required for regeneration.

## Competing interests

No author has any competing financial interest.

## Authors’ contribution

DC designed and supervised the study and drafted the manuscript, HL and WT carried out the experiments. HW gave advice for experimental design. TY gave advices of the experimental design, data interpretation and discussion, and edited the manuscript. All authors read and approved the final manuscript.
